# Mining key circadian biomarkers for major depressive disorder by integrating bioinformatics and machine learning

**DOI:** 10.18632/aging.205930

**Published:** 2024-06-13

**Authors:** Yuhe Shi, Jue Zhu, Chaowen Hou, Xiaoling Li, Qiaozhen Tong

**Affiliations:** 1 Department of Pharmacy, Hunan University of Chinese Medicine, Changsha, Hunan 410208, China

**Keywords:** major depressive disorder, circadian rhythm, bioinformatics, machine learning, biomarker, immune infiltration, drug prediction

## Abstract

Objective: This study aimed to identify key clock genes closely associated with major depressive disorder (MDD) using bioinformatics and machine learning approaches.

Methods: Gene expression data of 128 MDD patients and 64 healthy controls from blood samples were obtained. Differentially expressed were identified and weighted gene co-expression network analysis (WGCNA) was first performed to screen MDD-related key genes. These genes were then intersected with 1475 known circadian rhythm genes to identify circadian rhythm genes associated with MDD. Finally, multiple machine learning algorithms were applied for further selection, to determine the most critical 4 circadian rhythm biomarkers.

Results: Four key circadian rhythm genes (ABCC2, APP, HK2 and RORA) were identified that could effectively distinguish MDD samples from controls. These genes were significantly enriched in circadian pathways and showed strong correlations with immune cell infiltration. Drug target prediction suggested that small molecules like melatonin and escitalopram may target these circadian rhythm proteins.

Conclusion: This study revealed discovered 4 key circadian rhythm genes closely associated with MDD, which may serve as diagnostic biomarkers and therapeutic targets. The findings highlight the important roles of circadian disruptions in the pathogenesis of MDD, providing new insights for precision diagnosis and targeted treatment of MDD.

## INTRODUCTION

Major depressive disorder (MDD) is a prevalent and debilitating psychiatric condition characterized by persistent low mood, diminished interest, sleep disturbances, feelings of worthlessness, and recurrent thoughts of death [1–2]. According to the latest cross-national data, more than half of the global population can expect to develop one or more mental disorders by the age of 75 years, with similar rates between males and females. These disorders typically first emerge during childhood, adolescence, or young adulthood, with a peak incidence around age 15 years and a median age of onset of 19–20 years [3]. As a leading cause of disability worldwide, MDD imposes a substantial burden on individuals, families, and healthcare systems [4, 5]. Despite the availability of pharmacological and psychotherapeutic interventions, current treatments for MDD have significant limitations. Antidepressant medications, such as selective serotonin reuptake inhibitors and glutamate modulators, often have suboptimal efficacy, high relapse rates, and undesirable side effects [6, 7]. Cognitive-behavioral therapy, a common psychological intervention, also faces challenges in terms of patient accessibility and long-term effectiveness. Importantly, the underlying pathogenesis of MDD remains incompletely understood, hindering the development of more effective and personalized treatment strategies.

The multifactorial nature of MDD involves complex interactions between genetic, neurobiological, and environmental factors. Disturbances in neurotransmitters systems, signaling pathways, the hypothalamic-pituitary-adrenal (HPA) axis, and inflammatory responses have all been implicated in the pathogenesis of MDD [8]. Moreover, altered expression of biomarkers, such as brain-derived neurotrophic factor (BDNF), has been closely linked to the onset and progression of disorder [9]. Elucidating the clinical molecular characteristics of MDD is crucial for improving our understanding of its pathogenesis and enabling the development of novel diagnostic and therapeutic approaches.

Emerging evidence suggests that disruptions in circadian rhythms, the endogenous 24-hour cycles that regulate physiological processes, may play a pivotal role in the pathophysiology of MDD [10–12]. Patients with MDD commonly exhibit circadian rhythm disturbances, such as sleep abnormalities, disrupted temperature rhythms, endocrine dysregulation, and metabolic abnormalities [13]. These observations indicate that the biological clock governing circadian rhythms in MDD patients may be dysfunctional. Notably, some circadian genes, such as Brain and Muscle ARNT-Like 1 (BMAL1), have been demonstrated to significantly influence the onset and progression of MDD [14]. However, the specific roles of circadian mechanisms in MDD remain underexplored, and many potentially important circadian genes associated with the disorder are yet to be identified.

Importantly, this study adopts an integrative approach by combining advanced bioinformatics analysis and machine learning algorithms to systematically investigate the role of circadian rhythm disturbances in MDD [15]. Unlike previous studies that have primarily focused on individual circadian genes, our comprehensive analysis aims to identify novel key circadian biomarkers that can distinguish MDD patients from healthy controls. Moreover, we further explore the functional relevance of these circadian biomarkers in MDD pathogenesis, such as their associations with immune cell infiltration, and utilize computational drug discovery methods to predict potential therapeutic compounds targeting the key circadian proteins. The findings from this multilayered investigation are expected to provide new mechanistic insights into the circadian rhythm-MDD connection and pave the way for developing more effective diagnostic and treatment strategies for this debilitating psychiatric disorder.

## MATERIALS AND METHODS

### Data collection

Two gene expression profiles (GSE98793 and GSE76826) related to MDD were retrieved from the Gene Expression Omnibus database (GEO, https://www.ncbi.nlm.nih.gov/geo/). GSE98793 and GSE76826 were from GPL570 and GPL17077 platforms, respectively. The GSE98793 dataset included 128 patients with MDD and 64 healthy controls. The GSE76826 dataset included 12 patients with MDD and 12 healthy controls. MDD was determined by SIGH-D scores higher than 8. Gene expression in both datasets was from human blood samples.

In addition, a total of 1475 human circadian rhythm genes (CRGs) were obtained from the Circadian Gene Database (CGDB, http://cgdb.biocuckoo.org/), MSigDB database (https://www.gsea-msigdb.org/gsea/msigdb/) and Genecards database (https://www.genecards.org/) (Supplementary Table 1). These genes were used as the basis of this study.

In this study, GSE98793 was used as the training set for screening potential therapeutic targets for MDD and selecting circadian rhythm related key genes. The GSE76826 dataset was used as the validation set to verify the reliability of the prediction model.

### Identification of circadian rhythm genes (CRGs) related to MDD

#### Differential expression analysis

The GSE98793 dataset was used for differential gene expression analysis. First, the gene chip data in GSE98793 were normalized using the limma package in R software, and the genes were annotated. Differentially expressed genes (DEGs) between MDD patients and normal samples in the training set were screened with a threshold of *P*-value < 0.05.

#### Weighted gene co-expression network (WGCNA) analysis

We further analyzed the differential genes in MDD using WGCN analysis. Through WGCNA, functionally related gene co-expression modules were identified, and an unsigned co-expression network was constructed. First, clustering analysis of differential gene expression profiles was performed, and outliers were removed. Next, a “soft” threshold (β) value generated by the “pickSoftThreshold” algorithm was selected to construct an adjacency matrix and convert it to a topological overlap matrix (TOM). Then, high co-expression gene modules were generated by dynamic tree cutting. Finally, gene significance (GS) and module membership (MM) were calculated to determine the correlation between module feature genes (ME) and clinical traits. Differentially expressed genes that were closely related to the pathogenesis of MDD were screened in modules with GS >0.5, MM >0.5, and *P*-value < 0.05.

#### Identification of CRGs related to MDD

By intersecting the differential co-expressed genes obtained from the WGCNA network with the 1475 CRGs downloaded from the database, common genes were determined. These common genes were CRGs that influence the occurrence and development of MDD.

### Construction of protein-protein interaction (PPI) networks and functional enrichment analysis of CRGs

Protein-protein interaction (PPI) network analysis was performed for the CRGs related to MDD using the STRING online server (https://string-db.org/). Cytoscape 3.7.2 was used to visualize the results, and the degree parameters of the nodes in the network were analyzed.

To investigate the potential biological functions and signaling pathways of CRGs in the pathogenesis of MDD, functional enrichment analysis of MDD-related CRGs was performed using the online DAVID database (https://david.ncifcrf.gov/). Gene Ontology (GO) functional annotation and Kyoto Encyclopedia of Genes and Genomes (KEGG) functional enrichment analyses were applied for the analysis of MDD-related CRGs. The GO analysis covered three dimensions: biological processes (BP), cellular components (CC), and molecular functions (MF). It is a systematic method and process for annotating genes and their expression products. KEGG is an integrated database of genomic, chemical and systemic functional information, which is widely used for enrichment annotation of gene pathways. GO and KEGG analyses used a screening criterion of *P*-value < 0.05, and results were visualized.

### Identification of key CRGs related to MDD using machine learning

Three machine learning algorithms including least absolute shrinkage and selection operator (LASSO) logistic regression, support vector machine-recursive feature elimination (SVM-RFE), and random forest (RF) were utilized to identify key feature molecules among the differentially expressed CRGs in the GSE98793 database.

Key CRGs were selected based on LASSO model, SVM-RFE algorithm, and random forest model using R packages “rms”, “e1071”, and “randomForest”, respectively. Finally, the overlapping genes identified by the three machine learning algorithms were determined as key diagnostic circadian rhythm biomarkers with critical roles in predicting MDD.

### Validation of expression and ROC analysis for key CRGs

The expression levels of key CRGs in blood samples from MDD patients and normal controls were measured and verified by Wilcoxon rank sum test using the two datasets. First, the expression levels of key CRGs were determined in MDD patients and normal controls using the GSE98793 dataset as the training set. Then, the expression of these key CRGs was validated in the GSE76826 dataset.

To further test the accuracy of the key CRG selection in this study, and evaluate the diagnostic value of key CRGs as MDD biomarkers, receiver operating characteristic (ROC) curve analysis was performed on the GSE98793 and GSE76826 datasets using the “pROC” package in R.

### Immune infiltration analysis

CIBERSORTx (https://cibersortx.stanford.edu/), a computational tool based on single-cell RNA sequencing (scRNA-seq) data, can be utilized to infer the composition and relative proportion of immune cells in bulk tissues [16]. To further elucidate the relationship between MDD and immune cells, we uploaded the gene matrix data containing blood samples from depressed patients and normal controls to the CIBERSORTx database, and calculated the correlations between these genes and 22 types of immune cells. Wilcoxon rank sum test was used for analysis, and *p* < 0.05 was considered statistically significant. Subsequently, we also examined the distribution of potential key CRGs in immune cells as well as changes occurring in blood samples from normal controls versus depressed patients.

### Prediction of potential small molecule drugs

To uncover potential small molecule drugs that regulate key CRGs, we utilized Drug-Gene Interaction Database (DGIdb 4.0, http://dgidb.genome.wustl.edu/) and Connectivity Map (CMap) database (https://clue.io) simultaneously. DGIdb integrates drug-gene, drug-variant, and drug-tumor interactions from various public databases, enabling rapid discovery of potential drug targets and mechanisms of action [17]. CMap is a reliable, well-recognized genomics-based tool for discovering drugs to prevent diseases, which can predict drugs that may reverse or induce the expression of genes encoding a biological state through enrichment scores of positive and negative values [18]. We preferentially selected DGIdb results with higher-ranking scores and CMap database results with negative enrichment scores.

### Molecular docking validation

To validate the direct targeting interactions between key CRGs and predicted drugs, molecular docking was performed using Discovery Studio software (version 2019). First, three-dimensional structure files of key circadian rhythm target proteins and drugs were downloaded from the RCSB Protein Data Bank (PDB, http://www.rcsb.org/) and PubChem (https://pubchem.ncbi.nlm.nih.gov/) databases, respectively. Then, these protein and small molecule structures were imported into Discovery Studio software. To prepare for docking calculations, proteins and small molecules were preprocessed, including cleaning small molecule structures, adding partial charges, predicting small molecule protonation states to make them suitable for docking. Next, we defined the binding pocket regions of interest on the three-dimensional structures of the target proteins as the docking sites. Finally, LibDock in Discovery Studio was utilized for automatic molecular docking, which searches for the optimal binding modes between ligands and target proteins by rigid docking and inverse docking algorithms within the defined docking sites. After docking, LibDock scores were calculated for each ligand-target protein complex. Higher LibDock scores indicate stronger binding affinity between the ligand and target protein. Through this molecular docking process, we can evaluate the binding affinities between small molecule drugs candidates and the target proteins, thereby guiding drug optimization of drug design.

### Availability of data and materials

The datasets analyzed during the current study are available in the Gene Expression Omnibus repository (Accession Number: GSE98793 and GSE76826).

## RESULTS

### Identification of DEGs and WGCNA analysis

**Figure 1:**
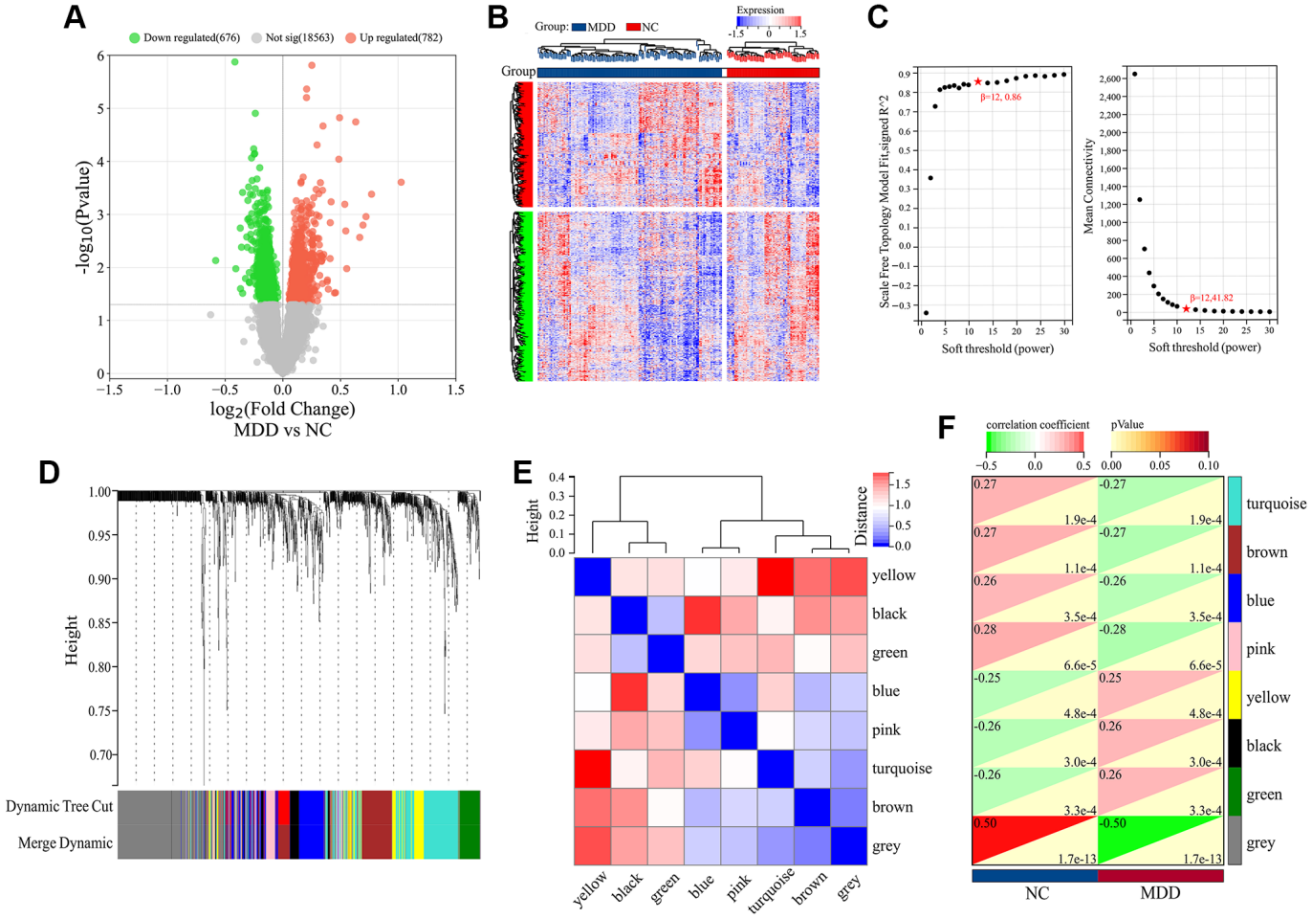
**Differential expression and WGCNA analysis of DEGs in GSE98793 dataset.** (**A**) Volcano plot of DEGs. (**B**) Hierarchical clustering of DEGs. (**C**) Scale independence and Mean connectivity as a function of soft-thresholding powers. (**D**) Cluster dendrogram. (**E**) Heatmap based on gene connectivity of modules. (**F**) Heatmap of clinical-trait associations.

**Figure 2:**
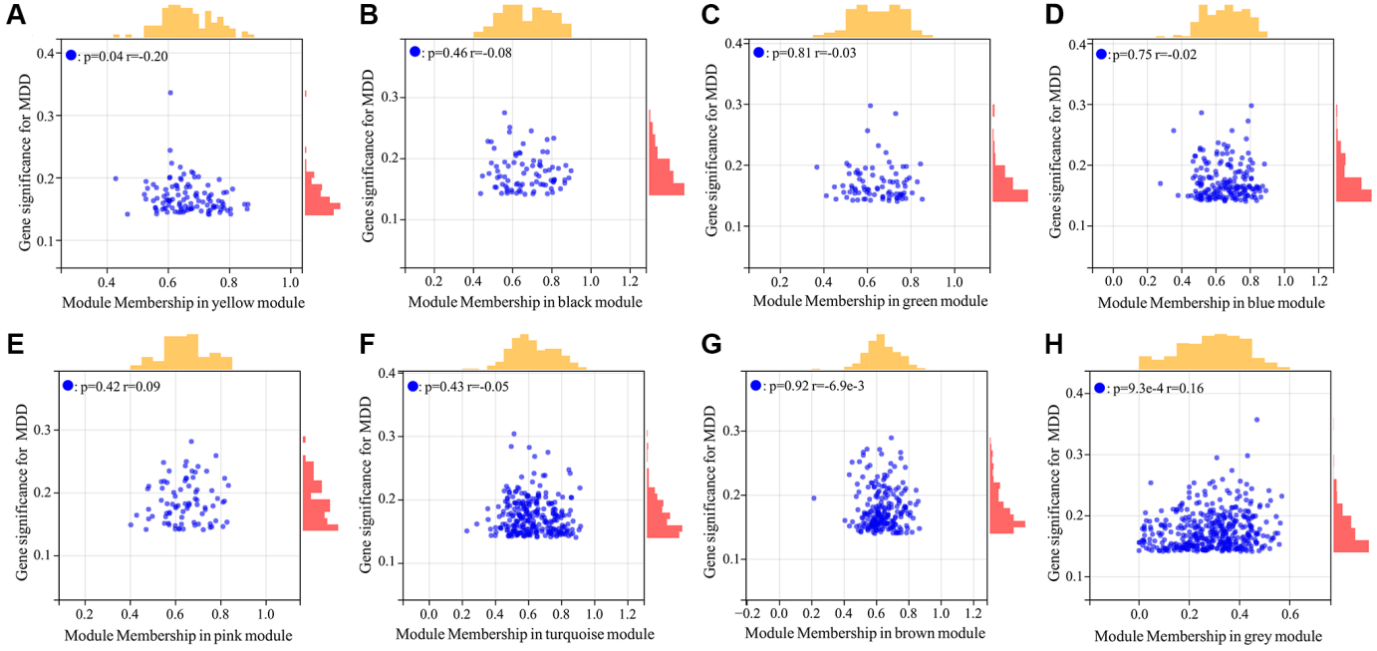
**Scatter plot analysis of hub genes highly associated with MDD from 8 co-expressed gene modules.** (**A**–**H**) Represented yellow, black, green, blue, pink, turquoise, brown, grey module respectively.

To explore genes associated with the occurrence and development of MDD, we first performed differential expression analysis on 64 normal samples and 128 MDD serum samples in GSE98793 dataset. The results showed that 1458 DEGs were identified between MDD patients and normal samples, including 676 down-regulated and 782 up-regulated genes (Figure 1A). The hierarchical clustering of DEGs was shown in Figure 1B. No abnormal samples were detected after clustering analysis with high threshold β of 12 (Figure 1C). Based on the dynamic tree cut algorithm, 8 gene modules were constructed with a minimum module size of 28, deep split of 4, and a maximum module distance of 0.25, including turquoise, brown, blue, pink, yellow, black, green and grey module (Figure 1D). Upon analyzing the connectivity of the module eigengenes (MEs), it was discovered that when the separation among modules exceeded 0.25, the genes within each module could operate independently (Figure 1E). By calculating the correlation coefficients between the modules and the clinical features of Major Depressive Disorder (MDD), it was found that all modules were significantly associated with MDD (Figure 1F). According to the criteria of |MM|>0.5 and |GS|>0.5, 954 genes highly correlated with MDD were identified from the 8 co-expressed gene modules (Figure 2). These hub genes exhibited expression patterns closely related to MDD phenotype, and were therefore defined as MDD-related targets.

### Screening of MDD-related CRGs

**Figure 3:**
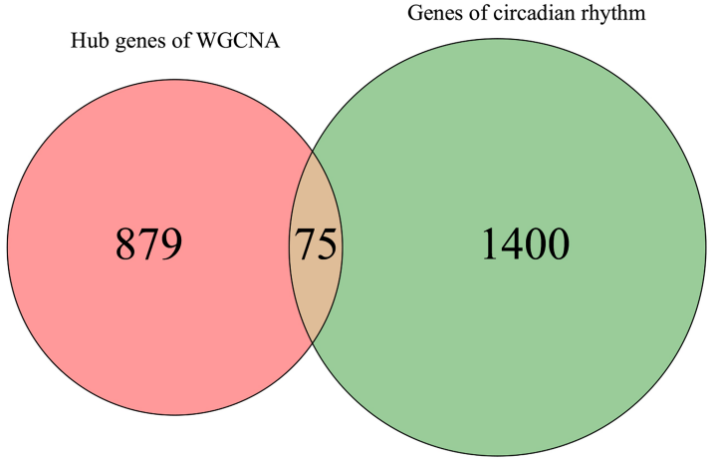
Venn diagram for screening MDD-related CRGs.

Given the well-established relationship between circadian rhythm dysregulation and MDD, we sought to identify the key circadian genes associated with MDD pathogenesis. By intersecting the 954 MDD-related targets identified in the previous step with a comprehensive list of 1475 known CRGs, we successfully identified 75 common targets between the two gene sets (Figure 3). These 75 overlapping genes were considered as the MDD-related CRGs, which may play crucial roles in the circadian rhythm disruptions underlying MDD development and progression.

### PPI network construction and functional enrichment analysis

**Figure 4:**
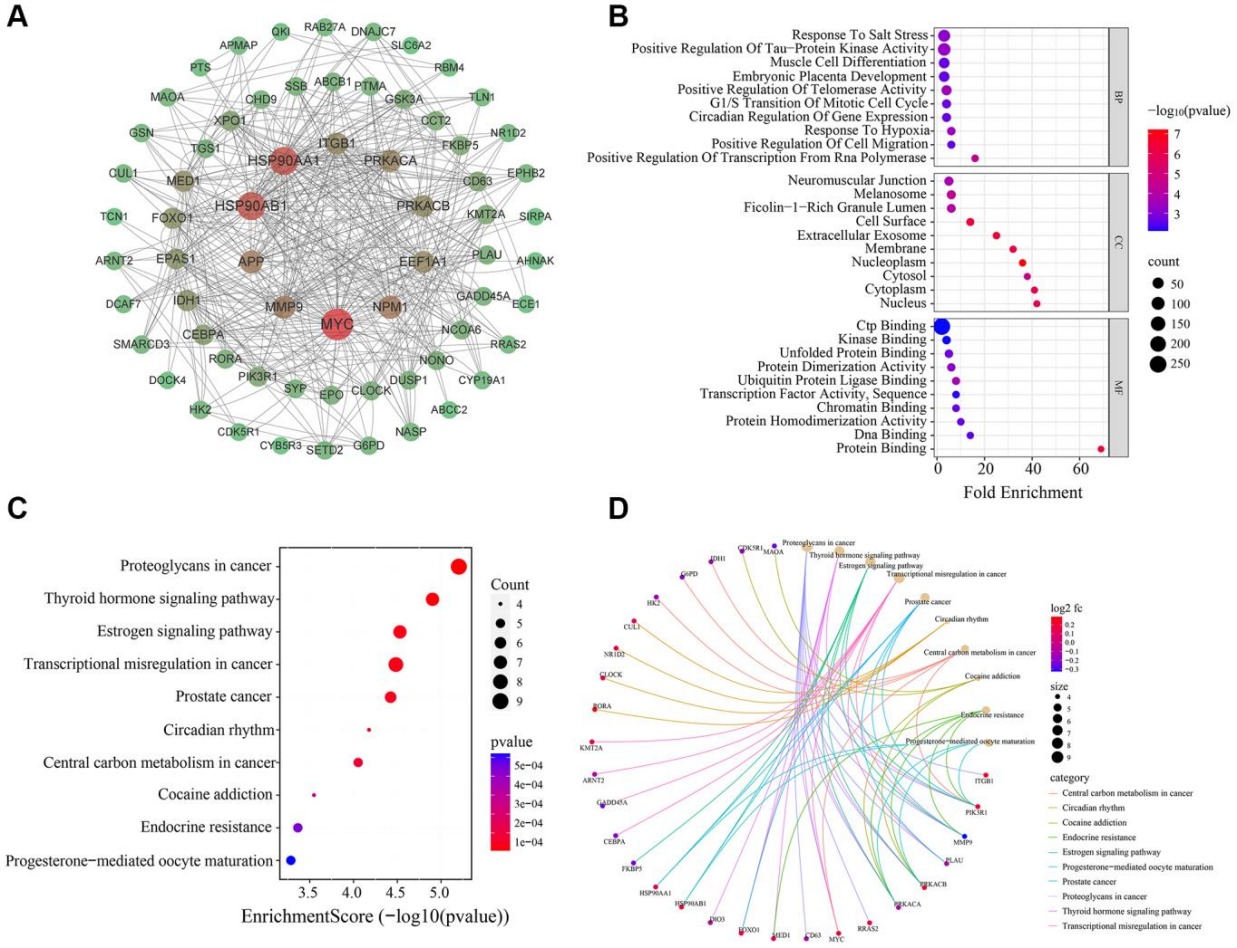
**Bioinformatics analysis of 75 MDD-related CRGs.** (**A**) PPI network of 75 MDD-related CRGs. Nodes represent targets and edges represent interactions. (**B**) Bubble chart of GO enrichment results. (**C**) Bubble chart of KEGG pathway enrichment results. (**D**) Enrichment plot of targets in pathways.

To gain deeper insights into the molecular mechanisms underlying the involvement of the 75 identified MDD-related CRGs, we constructed a protein-protein interaction (PPI) network using these genes (Figure 4A). The complex interconnections observed within this PPI network suggest that these CRGs may function in a coordinated manner to influence MDD pathogenesis.

To further elucidate the biological functions and pathways associated with these 75 MDD-related CRGs, we performed GO and KEGG enrichment analyses (Figure 4B–4D). The top 10 enriched GO terms covered a wide range of biological process, cellular component and molecular function. In biological process category, these CRGs were significantly enriched in transcription regulation, telomerase activity control, response to hypoxia, circadian regulation, and tau-protein kinase activity modulation. The cellular component analysis revealed the localization of these CRGs in neuromuscular junction, melanosome, cell surface and extracellular exosome. Regarding molecular function, the CRGs were found to be involved in CTP binding, kinase binding, unfolded protein binding, and protein activity.

The KEGG pathway analysis identified the top 10 significantly enriched pathways, including proteoglycans in cancer, thyroid hormone signaling pathway, estrogen signaling pathway, transcriptional misregulation in cancer, circadian rhythm, cocaine addiction, and endocrine resistance. These results suggest that the MDD-related CRGs identified in this study may play crucial roles in regulating diverse biological processes and signaling pathways, particularly those involved in circadian rhythm, neuroendocrine function, and cancer-related pathways, which are closely linked to MDD pathogenesis.

### Screening of key MDD-related CRGs using machine learning

**Figure 5:**
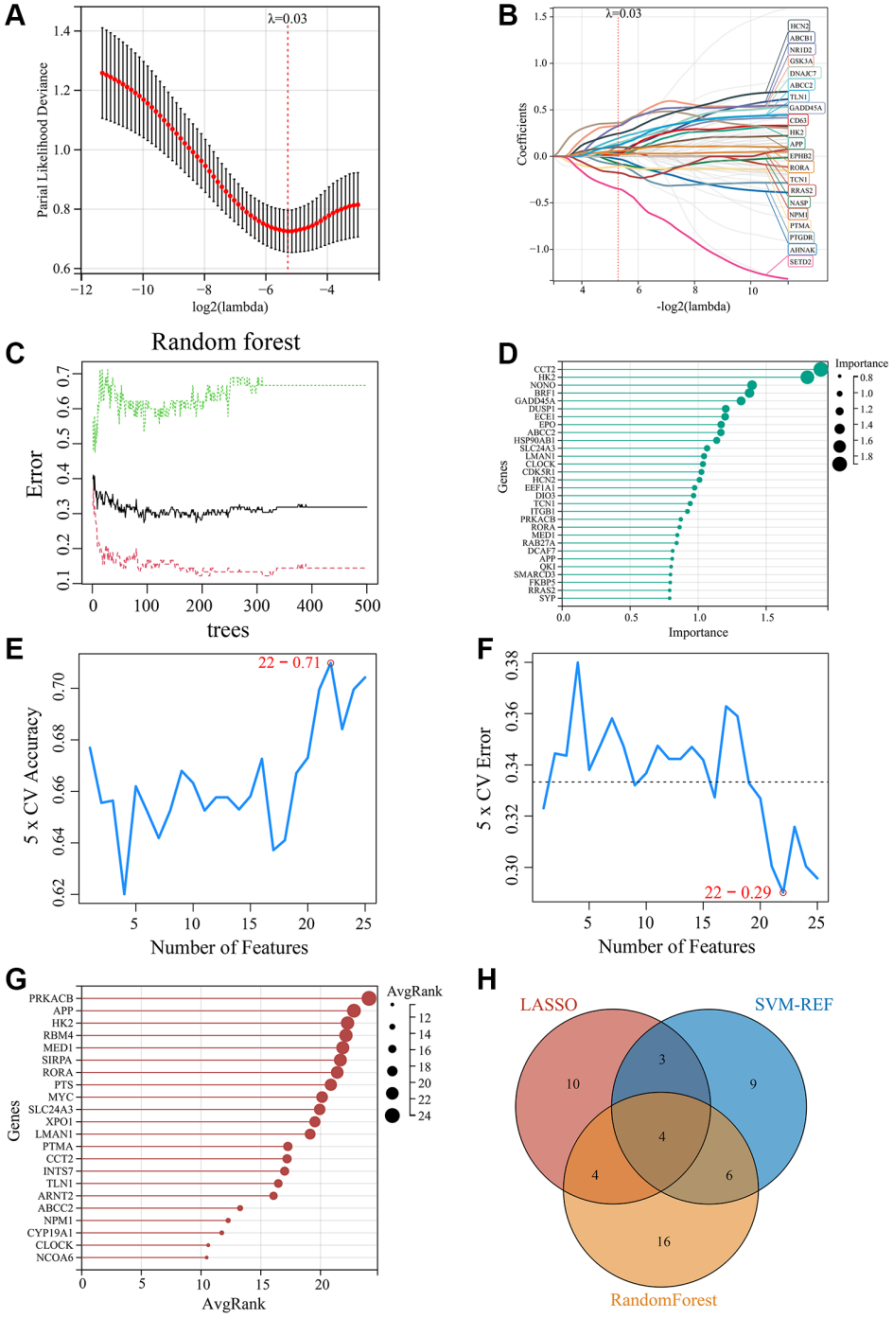
**Screening of key MDD-related CRGs using machine learning methods.** (**A**) Optimal λ selection for LASSO regression model. (**B**) LASSO coefficient profiles of the 21 feature genes. (**C**) Top 30 feature importance from random forest algorithm. (**D**) Top 30 important genes ranked by random forest algorithm. (**E**) Accuracy rate plot of SVM-RFE model. (**F**) Error rate plot of SVM-RFE model. (**G**) Top 22 genes with lowest error rate ranked by SVM-RFE. (**H**) Venn diagram for screening of the 4 key CRGs (ABCC1, APP, HK2, and RORA) by integrating LASSO, SVM-RFE and Random Forest algorithms.

To further identify the key CRGs most closely associated with MDD, we applied three machine learning methods - LASSO regression, SVM-RFE, and Random Forest - to the 75 MDD-related CRGs identified previously. First, we constructed a LASSO regression model to predict MDD status using the 75 CRGs. Ten-fold cross validation determined the optimal regularization parameter λ as 0.03 (Figure 5A). The LASSO model identified 21 feature genes that could accurately distinguish MDD samples from normal controls (Figure 5B). Next, we employed the Random Forest algorithm to rank the 75 CRGs by feature importance. The top 30 genes were selected as key feature genes (Figure 5C, 5D). We also performed SVM-RFE analysis, which identified 22 genes with good classification accuracy between MDD and normal samples (Figure 5E, 5F). These 22 genes were ranked by Avg rank (Figure 5G). By integrating the results of three machine learning algorithms, we finally identified 4 common key CRGs: ABCC1 (ATP-binding cassette subfamily C member 2), APP (Amyloid precursor protein), HK2 (Hexokinase 2), and RORA (RAR related orphan receptor A) (Figure 5H). These 4 key CRGs were considered the most promising potential biomarkers of circadian rhythm dysfunction in MDD patients.

### Expression analysis and dataset validation of key CRGs

**Figure 6:**
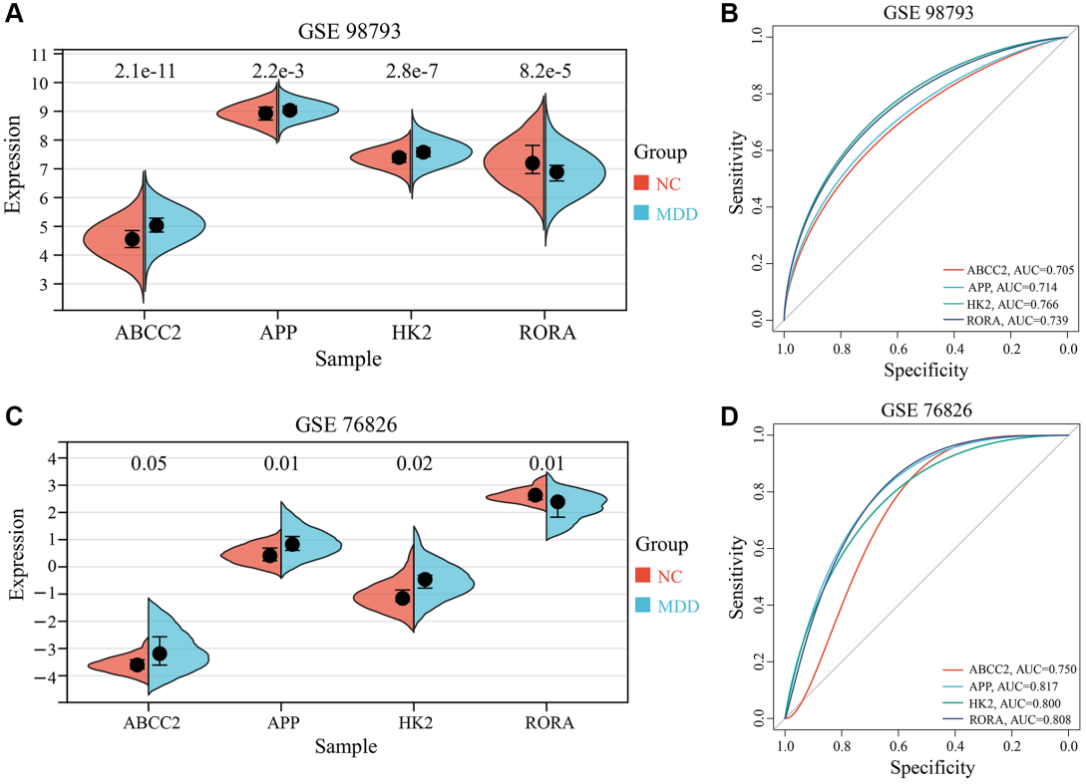
**Expression analysis and ROC curves of 4 key CRGs.** (**A**, **B**) Box plots and ROC curves of 4 key CRGs from training set (GSE98793). (**C**, **D**) Box plots and ROC curves of 4 key CRGs from validation set (GSE76826).

To validate the clinical relevance of the 4 key CRGs (ABCC2, APP, HK2, and RORA) identified in the previous section, we examined their expression in blood samples from MDD patients and healthy controls using the GSE98793 dataset as a training set and GSE76826 as an independent validation set. As illustrated in Figure 6A, 6C, three key CRGs - ABCC2, APP, and HK2 - exhibited significantly up-regulated in the blood samples of MDD patients relative to healthy controls, across both the training and validation datasets. In contrast, the expression of the circadian rhythm gene RORA was significantly down-regulated in MDD samples. To further evaluate the diagnostic potential of these 4 key CRGs as biomarkers for MDD-related circadian rhythm dysfunction, we performed receiver operating characteristic (ROC) curve analysis. As illustrated in Figure 6B, 6D, the area under the ROC curve (AUC) values for all 4 key CRGs were greater than 0.70 in both the training and validation datasets, suggesting they have good diagnostic accuracy in distinguishing MDD patients from healthy individuals.

### Analysis of immune cell infiltration

**Figure 7:**
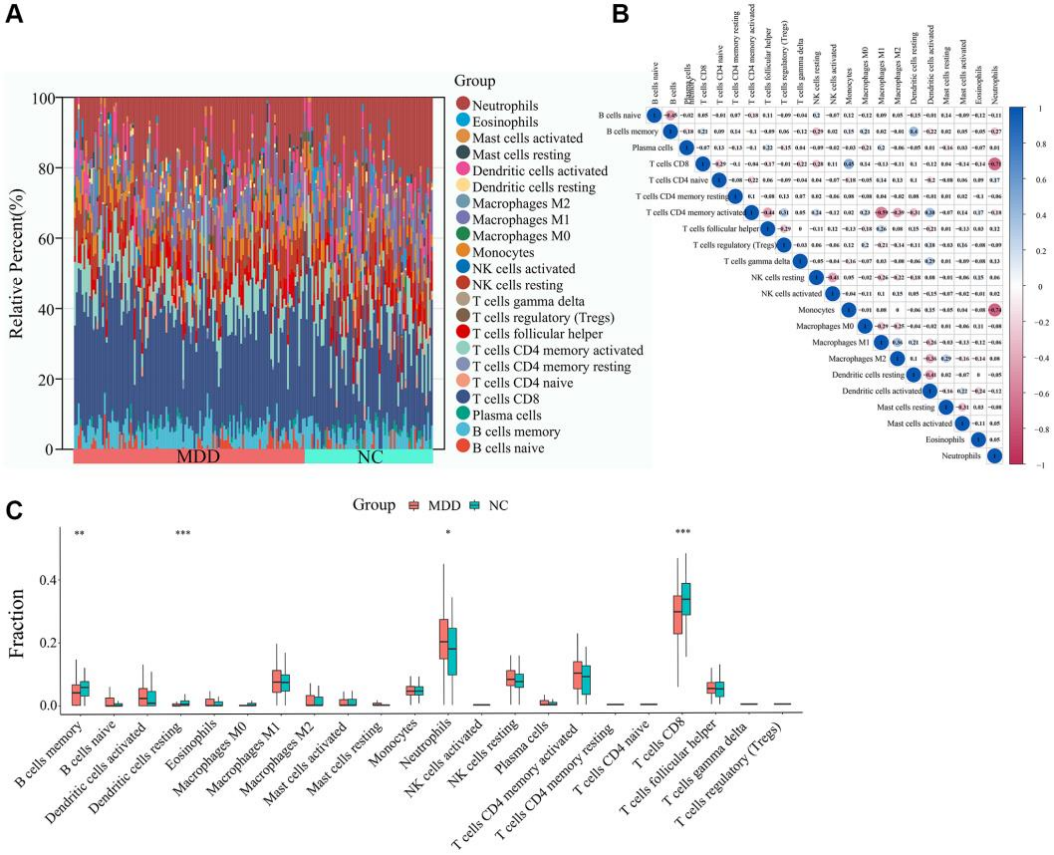
**Immune cell infiltration analysis.** (**A**) Abundance of immune cells in each sample. (**B**) Correlation analysis between immune cells. (**C**) Differential analysis of immune cell infiltration levels between MDD and normal blood samples.

To explore the potential involvement of immune dysregulation in MDD, we first compared the abundance of 22 different immune cell types between blood samples from MDD patients and healthy controls. As shown in Figure 7A, the immune cell composition profiles were distinct between the two groups. Correlation analysis of immune cell populations revealed several notable relationships (Figure 7B). Neutrophils were strongly negatively correlated with Monocytes (r = −0.74) and T cells CD8 (r = −0.71), while Macrophages M1 was positively correlated with T cells CD4 memory activated (r = 0.59). Further comparison of the immune cell infiltration levels between MDD and normal blood samples identified several significantly altered cell types (Figure 7C). Neutrophils were significantly increased, while B cells memory, T cells CD8, Dendritic cells resting were significantly decreased in the blood of MDD patients compared to healthy controls.

**Figure 8:**
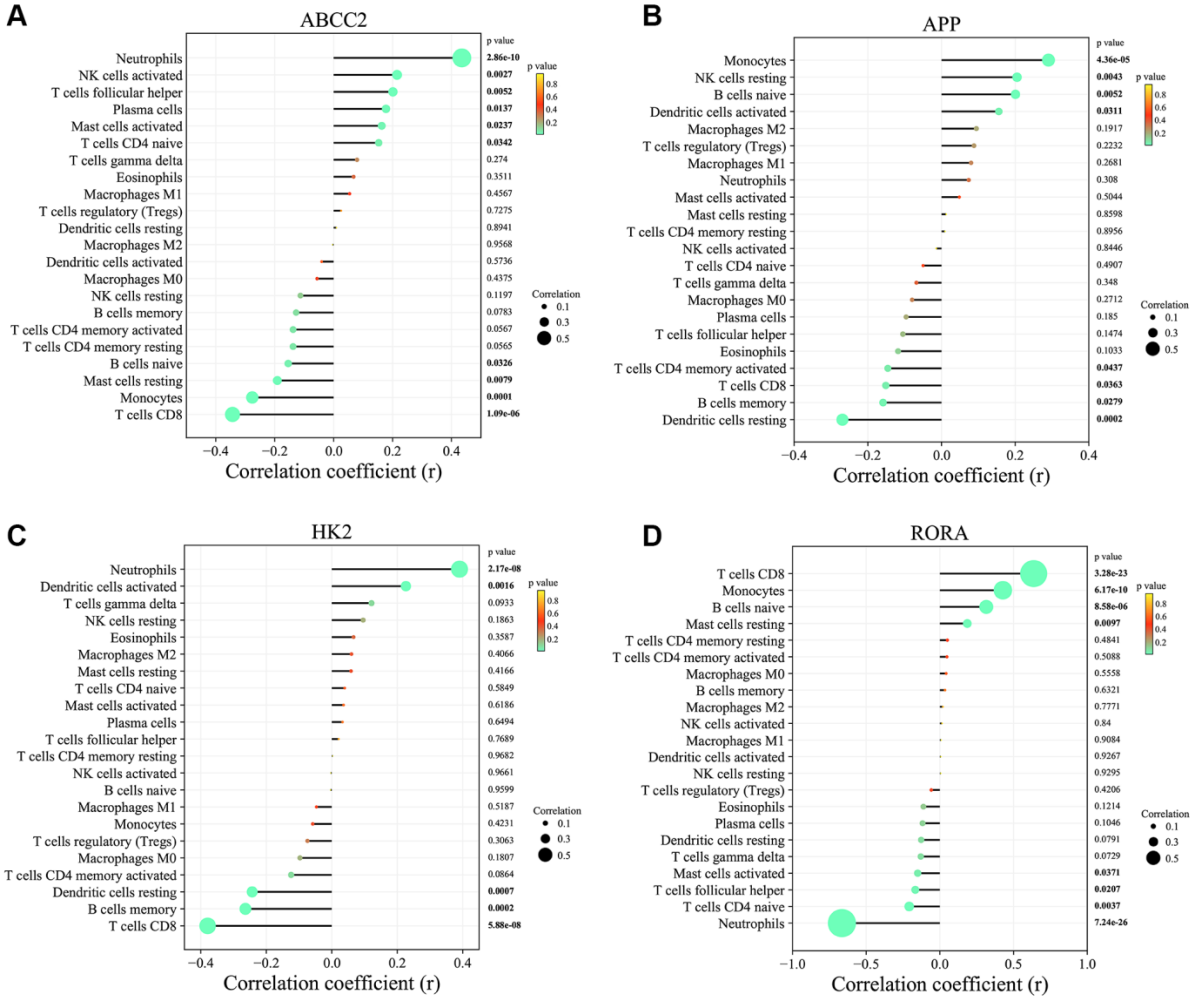
**Correlation analysis between key circadian rhythm genes and immune cell infiltration levels.** (**A**) ABCC2. (**B**) APP. (**C**) HK2. (**D**) RORA.

To investigate the potential links between the 4 key CRGs (ABCC2, APP, HK2, and RORA) and immune cell infiltration, we performed correlation analyses (Figure 8A–8D). ABCC2 was significantly positively correlated with Neutrophils and activated NK cells, but negatively correlated with T cells CD8 and Monocytes. APP showed positively correlated with Monocytes and resting NK cells, but negatively correlated with resting Dendritic cells and B cells memory. HK2 was positively associated with Neutrophils and activated NK cells, while negatively associated with T cells CD8 and B cells memory. Interestingly, RORA exhibited positive correlations with T cells CD8 and Monocytes, but negative correlations with Neutrophils and naive T cells CD4. These results suggest that the key CRGs identified in this study may be closely linked to the dysregulation of specific immune cell populations in MDD, which could contribute to the pathogenesis of the disease. Further investigation into the underlying mechanisms is warranted.

### Prediction of small molecule drugs and molecular docking

**Figure 9:**
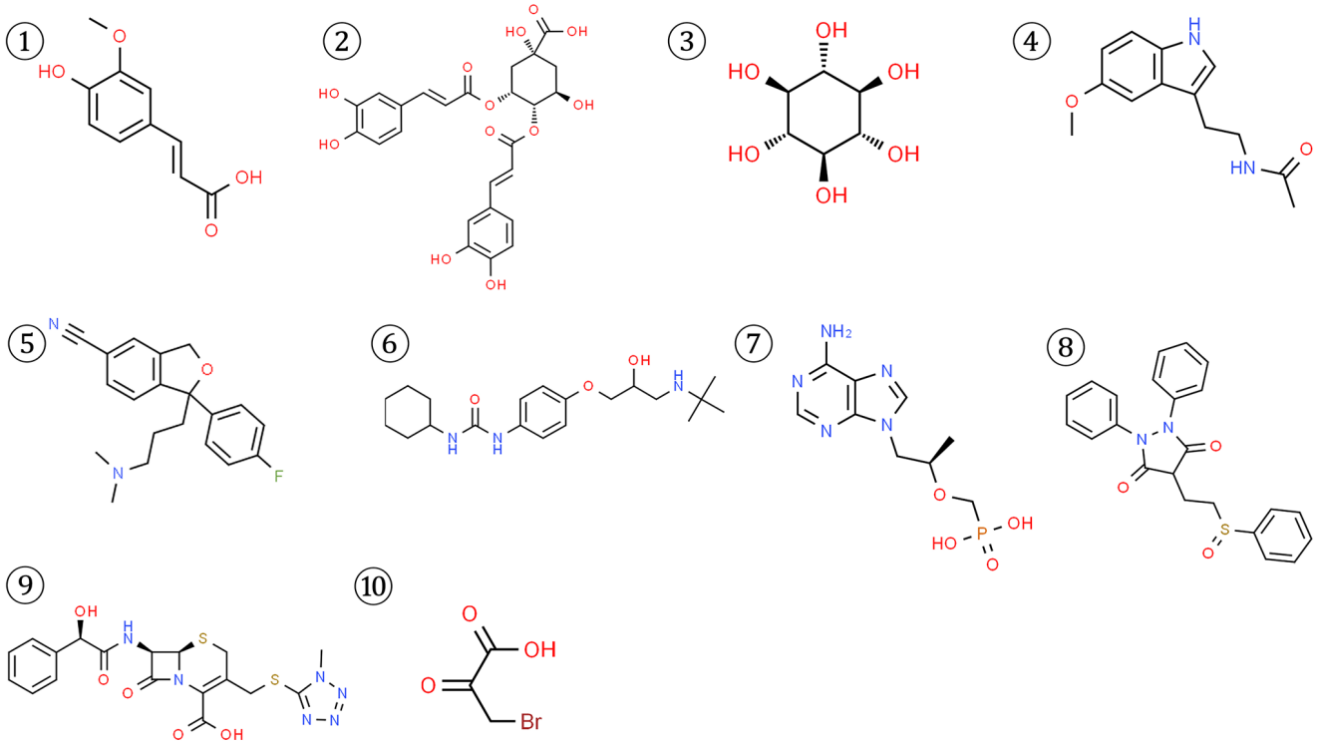
Structures of 10 potential small molecule drugs predicted to be associated with the 4 key CRGs from DGIdb and CMap databases.

To identify potential small molecule drugs that could modulate the imbalanced circadian rhythms associated with the 4 key CRGs (APP, RORA, ABCC2, and HK2), we queried the DGIdb and CMap databases. For APP, the top 3 related compounds from the DGIdb database were Ferulic acid, Isochlorogenic acid B, Scyllitol. For RORA, only 2 corresponding compounds were found-Melatonin and Citalopram. The DGIdb search for ABCC2 yielded 4 top-ranked compounds: Talinolol, Tenofovir, Sulfinpyrazone, Cefamandole. Additionally, 3-Bromopyruvic acid was the sole compound associated with HK2 in the CMAP database (Figure 9).

**Figure 10:**
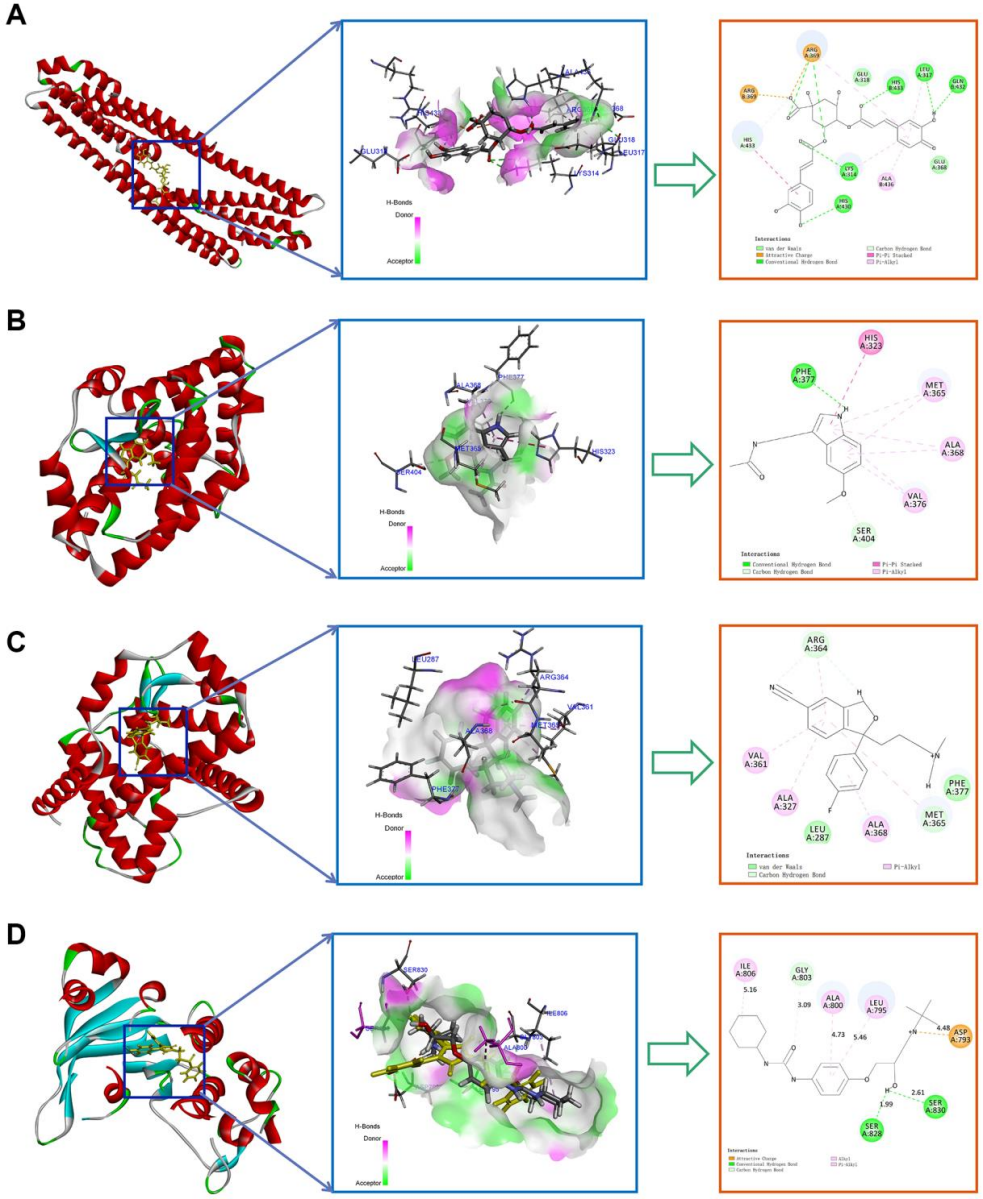
**Top molecular docking results for 4 key CRGs and potential drugs.** (**A**) Isochlorogenic acid B with APP. (**B**) Melatonin with RORA. (**C**) Citalopram with RORA. (**D**) Talinolol with ABCC2.

To further validate the potential interactions between these small molecule drugs and the 4 key CRGs, we performed molecular docking analysis. As shown in Table 1, APP, RORA, ABCC2 exhibited good docking interactions with their respective predicted compounds. Specifically, Isochlorogenic acid B had the tightest binding with APP (LibDock score of 126.536), Melatonin showed the highest docking value with RORA (125.850), and Cefamandole exhibited the highest affinity to ABCC2 (110.523). In contrast, 3-Bromopyruvic acid had a relatively low LibDock score of 53.369 with HK2, suggesting the need for further experimental verification of its interaction. The top 4 docking results are presented in Figure 10. These findings provide strong bioinformatics support for the potential of small molecule drugs to modulate the key circadian rhythm genes associated with MDD pathogenesis. Further in vitro and in vivo studies are warranted to validate the therapeutic implications of these drug-target interactions.

**Table 1:** Molecular docking results between 4 key CRGs and predicted drugs.

**Number**	**Name**	**Database**	**Target**	**Molecular formula**	**Interaction score**	**Libsore**
1	Ferulic acid	DGIdb	APP	C_10_H_10_O_4_	3.99	105.341
2	Isochlorogenic acid B	DGIdb	APP	C_25_H_24_O_12_	3.99	126.536
3	Scyllitol	DGIdb	APP	C_6_H_12_O_6_	3.99	67.178
4	Melatonin	DGIdb	RORA	C_13_H_16_N_2_O_2_	2.38	125.850
5	Citalopram	DGIdb	RORA	C_20_H_21_FN_2_O	1.25	111.481
6	Talinolol	DGIdb	ABCC2	C_20_H_33_N_3_O_3_	1.17	110.625
7	Tenofovir	DGIdb	ABCC2	C_9_H_14_N_5_O_4_P	0.97	110.189
8	Sulfinpyrazone	DGIdb	ABCC2	C_23_H_20_N_2_O_3_S	0.78	97.771
9	Cefamandole	DGIdb	ABCC2	C_18_H_18_N_6_O_5_S_2_	0.78	110.523
10	3-Bromopyruvic acid	cMAP	HK2	C_3_H_3_BrO_3_	−0.4368	53.369

## DISCUSSION

MDD is a debilitating mental illness with high mortality and disability. Recent epidemiological surveys reveal that the number of individuals suffering from MDD worldwide surpasses 350 million, with the COVID-19 pandemic markedly exacerbating the global burden of depression [19]. Emerging evidence has highlighted the significance of circadian rhythm abnormalities in the pathogenesis of MDD. Disruptions in sleep disorders patterns, diurna activity, hormone secretion, and other physiological processes regulated by the circadian clock represent core features of depressive disorders [11, 20–21]. While some CRGs located in the suprachiasmatic nucleus region of the hypothalamus, such as 5-hydroxytryptamine (5-HT), Basic helix-loop-helix ARNT-like protein 1 (BMAL1), Peroxidase 1-3 (Per 1-3), Nuclear receptor subfamily 1 group D member 1 (NR1D1), and D site-binding protein (DBP), have been observed in patients with MDD, the intricate functions of these CRGs pose formidable obstacles to their utilization as potential targets for the prevention, diagnosis, and treatment of the disorder [22, 23]. Fortuitously, the advent of bioinformatics and machine learning techniques has rendered the elucidation of disease pathogenic mechanisms and the identification of prospective disease biomarkers more feasible endeavors.

In the present study, we endeavored to synergistically leverage transcriptomic, bioinformatic, and machine learning approaches to explore potential CRGs biomarkers and investigate the influence of CRGs on the pathogenesis of MDD. Firstly, our findings revealed that several commonly disease-associated targets, such as RORA, Nuclear receptor subfamily 1 group D member 2 (NR1D2), Circadian locomotor output cycles protein kaput (CLOCK), and Cullin-1 (CUL1), were significantly enriched among CRGs within the circadian rhythm pathway. RORA can activate the expression of NR1D2, while NR1D2 in turn inhibits RORA, showing reciprocal regulation between them. Additionally, both RORA and NR1D2 can activate the transcription of circadian genes CLOCK, BMAL1, Cryptochrome-1 (CRY1) via ROM elements [24, 25]. As a transcriptional activator, CLOCK can activate the expression of downstream genes such as Per and Cry through the E-box (Enhancer element) element [26]. The CLOCK-BMAL1 complex can also activate the transcription of CUL1, promoting the degradation of circadian proteins [27]. The evidence presented above indicates that circadian rhythm disruption represents a crucial factor influencing the pathogenesis of depressive disorders. It is noteworthy that we found the circadian mechanisms in MDD might also be associated with cancer-related pathways and hormone-related pathways. MDD is a comorbidity of various cancers that significantly increases the risk of other unhealthy outcomes in patients [28]. One study has shown that prostate cancer patients are prone to develop depressive mood after surgery due to disrupted circadian rhythms [29]. Alterations in hormone and endocrine functions play an important role in the pathophysiological mechanisms of MDD. Currently, preliminary trials of hormone therapy have shown good preliminary results in MDD, including corticotropin releasing factor antagonists, glucocorticoid receptor antagonists, thyroid hormone-based HPT axis treatments, and estrogen replacement therapy of HPG axis [30].

To further identify potential circadian biomarkers associated with MDD, we employed three machine learning techniques to improve the efficiency and accuracy of biomarker screening. A panel of 4 key CRGs—ABCC2, APP, HK2, and RORA—were identified and validated that are strongly associated with MDD. ABCC2, APP and HK2 were significantly upregulated, while RORA was downregulated in blood of MDD patients. The consistent differential expression patterns and robust diagnostic performance of these CRGs across two independent blood transcriptomic datasets provide compelling evidence for their potential utility as biomarkers of MDD-related circadian rhythm disturbances. ABCC2, also known as multidrug resistance-associated protein 2 (MRP2), belongs to the ATP-binding cassette (ABC) transporter family, participating in the transport of drugs and pathogenic metabolites [31]. It has been found that the expression and function of ABCC2 exhibit circadian rhythmic patterns, the transcription of which is inhibited by Rev-erbα, a downstream gene of BMAL1/CLOCK [32]. Existing studies have demonstrated that the function of ABCC2 impacts the pharmacokinetics and pharmacodynamics of antidepressant medications, thereby influencing therapeutic outcomes [33]. ABCC2 may be implicated in the pathogenesis of MDD through its involvement in the transport of neurotransmitters and hormones across the blood-brain barrier. For instance, elevated plasma levels of 5-HT, a crucial antidepressant neurotransmitter, have been demonstrated to suppress ABCC2 expression [34]. This finding corroborates the trend observed in our research results. Moreover, ABCC2 also regulates the transport of glucocorticoids, which are critical mediators of the stress response and intimately associated with the onset and progression of depressive disorders [35]. APP is a transmembrane protein mainly located in synaptic regions of neurons, involved in regulating synapse formation, neurotrophy and neuroplasticity. The participation of APP in circadian mechanisms is associated with disruption of CLOCK/BMAL1 regulatory elements, interference with circadian gene expression, and disturbance of circadian rhythms [36, 37]. It is well-established that aberrant splicing and metabolism of the APP, leading to the production of beta-amyloid, constitutes the critical pathological basis for Alzheimer’s disease. An accumulating body of evidence suggests the existence of a shared genetic foundation between MDD and Alzheimer’s disease [38, 39]. These findings illustrate that APP also plays a pivotal role in the pathogenic mechanisms underlying depressive disorders. On one hand, aberrant APP metabolism and amyloid aggregation can induce neuronal injury and apoptosis, which is consistent with the hippocampal and cortical atrophy observed in patients with MDD [40]. On the other hand, APP also influences the expression of proteins associated with neuroplasticity, such as brain-derived neurotrophic factor (BDNF) and Postsynaptic density protein 95 (PSD-95), thereby disrupting synaptic remodeling, which may represent a critical molecular event in the onset of depressive episodes [41]. HK2 is a rate-limiting enzyme that catalyzes the phosphorylation of glucose and plays a key role in glycolysis. The involvement of HK2 in circadian mechanisms is associated with effects on glucose metabolism in the body. Singh Gurjit et al. found that HK2 in the brain of North American wood frogs exhibited circadian rhythmic expression changes, which was related to the regulation by the bHLH family transcription factor MondoA-MLX complex [42]. This study provided clues on the rhythm of biological clock and metabolism. Another study showed that specific deletion of the circadian gene BMAL1 would affect mRNA expression and activities of HK2 and phosphofructokinase 1 (PFK1) in tissues, further influencing systemic glucose homeostasis [43]. The above studies demonstrated the key evidence of HK2 in circadian rhythms and glucose metabolism in the body. The RORA gene encodes the retinoic acid-related orphan receptor alpha (RORα), a member of the nuclear receptor superfamily. RORα plays a crucial role in regulating various physiological processes, including circadian rhythms, energy homeostasis, neurodevelopment, and immune function. Both genetic polymorphisms in RORA and alterations in its expression levels have been closely associated with an increased susceptibility to depressive disorders [44–46]. As a core clock protein, functional disruptions in RORα can directly impair the regulation of circadian rhythms, thereby increasing the risk for developing depressive disorders [47–49]. Moreover, RORα participates in the regulation of neurogenesis and neuroplasticity through modulating the expression of crucial molecules such as BDNF and 5-HT, processes that are intimately linked to the neurobiological underpinnings of MDD [50, 51]. Recent studies have also uncovered a potential role for RORα in influencing inflammation-associated processes relevant to MDD through its immunomodulatory functions [52, 53]. In summary, the four genes ABCC2, APP, HK2, and RORA are respectively involved in crucial molecular events and physiological processes intimately linked to the pathogenesis of depressive disorders, including neurotransmitter transport, amyloid metabolism, energy dysregulation, and circadian disruption. Their aberrant expression patterns provide potential biomarkers for the precise diagnosis of depression and evaluation of its severity. A comprehensive understanding of the multifaceted roles played by these biomarkers in governing diurnal rhythmicity, neuroplasticity, and mood homeostasis will shed light on the underlying biological pathways implicated in depressive symptomatology and inform the development of integrated therapeutic approaches.

Notably, the analysis of immune cell infiltration in blood samples revealed distinct alterations in MDD patients compared to healthy controls. This finding was consistent with a previous report in the field [52]. Specifically, Neutrophils were significantly increased, while memory B cells, CD8+ T cells, and resting Dendritic cells were significantly decreased in MDD patients. The increased infiltration of Neutrophils, a pro-inflammatory cell type, in MDD blood samples is consistent with the growing body of evidence linking neuroinflammation and immune dysregulation to the pathogenesis of depressive disorders [53]. Conversely, the reduced levels of memory B cells, CD8+ T cells, and resting Dendritic cells suggest a potential impairment in adaptive immune responses and antigen presentation in MDD patients [54, 55]. These findings align with previous reports of altered lymphocyte and antigen-presenting cell populations in depression [56]. Importantly, the correlation analyses between the 4 key CRGs and the infiltration levels of immune cell populations provide valuable insights into the potential mechanisms linking circadian rhythm disturbances and immune dysregulation in MDD. For instance, the strong positive correlation between RORA and the infiltration of CD8+ T cells and Monocytes suggests that RORA may play a pivotal role in regulating the anti-inflammatory functions of these immune cell types [57, 58]. Conversely, the negative correlations between RORA and Neutrophils, as well as naive CD4+ T cells, indicate that disruption of RORA-mediated circadian control could contribute to the pro-inflammatory state observed in MDD.

Importantly, the observed correlations between the 4 key CRGs and the infiltration levels of specific immune cell populations, such as neutrophils, T cells, and NK cells, suggest a potential mechanistic link between circadian rhythm disruption and immune dysregulation in MDD. This finding is consistent with growing evidence supporting the critical role of neuroimmune system imbalances in the development and progression of depressive disorders [59]. However, in contrast to previous studies that have primarily focused on individual circadian genes or immune markers [60, 61], the present work has uniquely identified a panel of CRGs that are closely associated with both circadian and immune perturbations in MDD. Similarly, the positive associations between ABCC2, APP, and HK2 with the infiltration of Neutrophils and activated NK cells, coupled with the negative correlations with CD8+ T cells and Monocytes, imply that dysregulation of these CRGs may impair the balance between pro-inflammatory and anti-inflammatory immune responses in MDD. These findings build upon the growing body of evidence highlighting the critical role of the brain-immune axis in the pathophysiology of depressive disorders [62]. The present study’s identification of a panel of key CRGs and their associations with specific immune cell populations represents a significant advancement compared to previous research that has primarily focused on individual circadian genes or immune markers. These findings provide a more comprehensive understanding of the complex interplay between disrupted circadian rhythms and immune dysregulation in MDD, and offer potential targets for the development of novel diagnostic and therapeutic strategies.

Most available antidepressants nowadays work through monoamine mechanisms [63]. To effectively explore drugs targeting key CRGs, this study performed screening, prediction and analysis of antidepressant drugs for 4 key CRGs, and obtained some referable results including melatonin and citalopram. Melatonin or melatonin receptor agonists are currently the main and effective drugs for regulating circadian rhythm disorders in MDD [64]. Agomelatine, the first approved antidepressant targeting melatonin receptors, significantly improved sleep quality and reduced waking in patients [65]. Citalopram, a selective serotonin reuptake inhibitor, is increasingly used for MDD treatment. It has the advantage of a rapid onset of action and exerts a significant antidepressant effect by modulating multiple neurotransmitters, including dopamine, GABA, and norepinephrine [66, 67]. In addition, we also screened some natural products from plants such as ferulic acid and isochlorogenic acid B. Ferulic acid has been proven to produce significant antidepressant effects in animal models of MDD through various mechanisms, providing a solid basis for its clinical application [68]. Ferulic acid can significantly improve APP deposition-induced neurofunctional impairments by activating the PI3K/Akt signaling pathway [69]. Isochlorogenic acid B, abundant in plants like honeysuckle, chrysanthemum leaves, propolis, was found to have good effects on improving MDD and neuroinflammation. Its mechanism is associated with regulating brain-derived neurotrophic factor signaling pathways [70]. More data are needed to support the direct effects of alternative drugs on MDD.

In summary, this integrative study revealed four key circadian rhythm genes (ABCC2, APP, HK2, and RORA) that are closely associated with MDD pathogenesis and may serve as promising diagnostic biomarkers and therapeutic targets. The identified genes were found to be functionally involved in circadian rhythm regulation, neuronal processes, immune responses, and metabolic pathways implicated in MDD. Additionally, these genes showed significant correlations with immune cell infiltration profiles, highlighting the potential contribution of circadian rhythm disruption and neuroinflammation to MDD etiology. Furthermore, we predicted several small molecule compounds that could directly target these circadian proteins, providing a foundation for future drug development efforts. Collectively, our findings shed new light on the intricate relationships between circadian rhythms, immune dysregulation, and MDD pathophysiology, paving the way for more effective diagnosis, prevention, and treatment strategies for this debilitating disorder.

Looking ahead, further experimental validation of the identified circadian biomarkers and their functional roles in MDD pathogenesis is warranted. Additionally, preclinical and clinical studies are needed to evaluate the therapeutic potential of the predicted circadian rhythm-modulating compounds for MDD treatment. Given the multifaceted nature of MDD, a combinatorial approach targeting multiple circadian genes and pathways may be required for optimal therapeutic efficacy. Furthermore, integration of circadian rhythm monitoring and chronotherapeutic interventions into existing treatment paradigms could enhance the personalization and precision of MDD management. By elucidating the complex interplay between circadian rhythms, neurobiological processes, and environmental factors in MDD, we can pave the way for more holistic and tailored strategies to alleviate the substantial burden imposed by this prevalent mental health disorder.

## Supplementary Materials

Supplementary Table 1
