# Supplementary material for: Mining key circadian biomarkers for major depressive disorder by integrating bioinformatics and machine learning
Source: Aging (Albany NY). 2024 Jun 13;16(12):10299–320. doi: 10.18632/aging.205930 (PMC11236317; doi:10.18632/aging.205930)
Supplement: Supplementary Table 1 [file aging-16-205930-s001.pdf]

SUPPLEMENTARY TABLE

Supplementary Table 1. 1475 circadian rhythm genes (CRGs) collected from the MSigDB database.

SLC2A2	SUMO3	SYNCRIP	CCT7	CCNE1	CSMD1	PPARGC1A	DDIT3	CBL	CREB3L3	KMT2D	PI4KA
RBP2	GPR176	PDZD3	SSB	DICER1	ATM	TNFRSF11A	ATF3	TSC2	TRH	NAT2	SLC2A3
MEF2D	PSMC4	AVPR1A	RAB5B	PEMT	CYP2E1	EPHB2	CEBPG	SPRY2	DGKQ	DIO3	ADCY3
CLOCK	ATF2	MAX	SMCHD1	RGS20	VRK2	PTCH1	NPAS3	GATD3	MPO	ICAM1	APRT
PER2	APP	CRTC2	TAGLN2	PGR	PSMB1	NEUROD1	IL4	CYP3A4	SULT2A1	CAVIN2	BCKDK
PER3	FDFT1	CRTC3	RAB1A	XPA	PSMD12	TRIM35	PNPLA3	MED9	TELO2	SULT1A1	CYP7B1
PER1	PDE6B	TDRKH	HNRNPH3	SLC12A2	RHOA	NCOR2	USP30	SEM1	EHMT2	FABP4	ENO2
CRY1	SUGP1	RGS16	AHNAK	SLC5A1	GSR	MYBBP1A	DDC	BRINP1	CDK6	IL6R	HPRT1
ARNTL	THBS1	GNAQ	EIF3E	NUP98	OLFML3	CSNK1A1L	MEF2A	BLMH	IKBKB	ADRA2C	PDK1
CRY2	ACADS	TH	RALY	RAE1	RB1	MIF	GUSB	GRM5	TPO	UCP2	SUCLA2
TIMELESS	CLTC	H2BC11	AURKAIP1	NPSR1	GCK	HTR1B	KDM1A	ARID1A	NMNAT1	WEE1	ATIC
NPAS2	PLCB4	SUV39H1	APMAP	HELLS	ABCB7	CALCA	SRD5A1	RABGEF1	ABCC3	EGFR	CTPS1
ARNTL2	GLO1	PRMT1	MAGEC1	IMMT	ANGPTL2	MAPK3	DRD3	RAP1GAP	PKLR	CYP1A1	FDXR
CIPC	TIPIN	RPS19	CPNE9	SUV39H2	SNCA	TRIP12	PTGDR	KCNQ1	INPP5D	CALB2	GNAI1
CSNK1D	PPP2R1B	HMGA1	HID1	CHI3L1	JAK2	TERT	ARRB1	FOSB	PFKP	HDC	GNAI2
NR1D1	PRKDC	RPL36	SEPTIN7	MAFK	NR1H3	TNPO1	CYP11B2	GADD45A	PIK3R5	CDK2	GPT2
CSNK1E	PRNP	RPL39	ADAR	APC	ESRRB	PRKN	MRE11	TUBA1B	ACSF3	ARF1	HSD17B4
CIART	SREBF1	APOE	USF1	TF	CAMK1	LEMD3	GLUL	ABHD5	IRF4	GUCA2B	ITPR3
BHLHE40	ENOX2	KAT8	ERN1	MMP3	NCAM1	UCHL5	PIK3C2A	HS3ST2	MECR	DUSP4	RAD21
BHLHE41	SLC41A1	ADRB1	CDKN2A	COPA	ATG14	TBL3	PPOX	XBP1	PANK2	CYP1B1	SMC3
RORA	TBL1X	CBX3	UBR5	SERPINF2	H2BC15	UBE2O	PCNA	NFE2	HAS3	FADS1	CHD2
DBP	GRP	GPI	ZBTB17	STAR	AQP3	COPS6	BAX	HMGCR	B4GALT2	CDC25A	ENPP2
PASD1	PPAT	LEPR	ATG7	MITF	SPSB1	ZNF207	ADRB2	ANKK1	CIDEC	JUNB	PCBD1
NOCT	XPO1	ZNF44	TRAF2	GSTP1	RPA2	LOC102724428	XDH	HEBP1	FFAR1	FBXO31	PFKFB3
AANAT	SPR	PSME2	ORMDL3	PIK3R2	MAOA	ZNF174	RPL3	CYP11B1	BRWD1	CLSPN	VAPB
NR1D2	HAT1	METTL14	CES1	PEBP1	FHIT	USP38	PSME4	FABP7	SERINC1	EGF	CTBP2
FBXL3	RACK1	COPS2	BGLAP	ABCB1	CHRM3	OGG1	HUS1	DCP2	DHX29	FBXL14	EBP
SIRT1	TGS1	MAPK14	HES1	GIP	NECTIN1	KCNC2	PRDX6	SIM1	SLC25A27	ADK	EMD
NFIL3	CHD9	CS	NPY2R	EPO	THPO	AHRR	SYP	MT2A	SRPRB	CYP17A1	GATAD2B
RAI1	HELZ2	PPP2R5D	HRAS	IFNA1	RYR2	CDKN1B	IGF1R	CHD4	FUT5	FBP1	HDAC10
GSK3B	RASD1	HSPA5	AGRP	SLC9A3	AR	PBRM1	RRAS2	CEBPE	KCNG2	IDS	PI4KB
OPN4	KITLG	ZPBP2	SHBG	PPP2CA	GGT1	CST3	EPB41	MTAP	YTHDC1	CACNA1A	PTPN13
BTRC	HBB	HSF1	CDKN1A	ATR	NR1I2	ADORA2A	GRM7	AFP	EXOSC4	GLDC	SDC4
NR3C1	EPAS1	P4HB	RPS2	MT1F	NR6A1	PRKG1	HABP2	GAPVD1	ENY2	ACACB	SDHD
CREBBP	NFKB2	ALDOA	ARNT2	FOXP3	MAGEC2	STX1A	PRKCB	RHOD	H1-5	AK1	GNB2
CSNK2A1	LRPPRC	TLN1	ADCY1	TLR4	TAC1	TAB2	TRPA1	ITGB1	KMT5B	AKR1B1	GNB4
HDAC3	HSPA8	PDIA6	NAGLU	SLC6A3	GPT	GFRA1	CBLB	PRKCD	MTARC2	HADHB	SULT1A2
TP53	DNAJA1	HSPA1A	NOS1	HBA1	SLC46A1	PURA	FANCL	ADSL	DUSP6	LBR	TINF2
PPARA	MAGED1	FOXO1	MAP2K7	CELF2	FGF21	EIF4G2	GRIK1	ALDH6A1	CXCL8	ADD1	BAZ1B
ID2	WDR5	NCL	PRKAA2	CNP	RPS6KA1	MYF6	PTPRT	ADCY8	HSPA4	RBP4	CUL7
EP300	AFF2	SST	SLC2A1	TUBB	DNAH8	PRKAR1A	QKI	CA14	HTR3A	SULT2B1	EBF1
NCOA2	CEBPA	CCL2	SLC16A1	YWHAB	CCL4	BTG1	ATRIP	CYP51A1	H3-3B	PDK4	GBA2
CREB1	BDNF	HK1	TMPO	YWHAQ	IGFBP7	CLDN5	COL9A1	MANBA	LPL	AADAC	GGT7
CRTC1	ATOH7	LMNA	HTT	DLGAP1	ADIPOR1	SNRNP200	TOB1	CCS	ODC1	ADH7	SLC3A2
PROK2	APOA1	PRKD1	HNRNPK	HNRNPH1	ADIPOR2	LDHD	DSCAM	AK5	PIK3CG	DDAH1	ST3GAL4
PPP1CA	VCP	PGK1	PRDX2	NASP	JUND	NCKAP1	NTM	SLC25A17	SSBP1	MAOB	BAZ1A
PPP1CB	FASN	ACACA	PSMC2	ETV6	PSME1	NCOA4	OPHN1	BRMS1	INTS2	DIO1	CDC16
PPP1CC	YWHAE	MDH2	ATF1	STAT3	PIGF	HLA-DQB1	RGS6	DNAJC2	GBA	EXOSC2	DDHD2
NPY	PAICS	ENO1	VRK3	HTR2C	CYP19A1	RBPMS	RND3	PTGR2	OPRM1	EXOSC3	HMGN2
NRIP1	SIN3A	HADHA	HBA2	CGA	CAT	ZFR	DOCK4	ERI1	MC4R	FBP2	CTPS2
RORC	GFPT1	HSP90B1	SRC	GAST	PDE4D	DAZAP2	SEC23IP	NUDT3	HPGDS	IFNB1	MTMR6
SERPINE1	GNB3	NME2	PRDX1	UCP3	EYA1	STBD1	ANKS1B	PPM1L	BAD	VAMP2	RNF40
THRAP3	DRD4	PCCA	RANBP2	ATP1A1	OPCML	GTDC1	IRX1	COQ10B	SCN1A	OSBP	HNRNPA0
MTNR1A	APOB	PCCB	RPL10	NPM1	MYRIP	APOH	LRRC4C	YIF1A	NR5A2	AOC1	MMS19
EZH2	MATR3	VDAC1	RUVBL1	ATP1A3	CUL3	GRM1	RNF19A	SPTSSB	KIF11	EXOSC5	MT1A
RBM4	YTHDF2	CYB5R3	RUVBL2	CRKL	SERPINA7	MMADHC	SORCS1	HIKESHI	PDE4A	TNRC6A	ABHD14B
KDM2A	ADORA1	SLC10A2	RPS14	MCM4	GNA11	HNRNPA2B1	ATP10B	MMP9	TRPV6	GJA1	ATP6V0B
KMT2A	GPR50	HSD17B10	RPS17	TUFM	FBXL17	ST3GAL1	CD180	NRARP	TRPV5	MCL1	GLYATL1
USP2	EXOC1	NME1	PSMC1	YWHAH	SKP2	ING3	FOXN3	ZNF423	GRIK2	BRD4	AGMO
KDM5A	YWHAZ	PRKD3	WDR77	EEF1A2	TGFB2	ITPK1	NAALADL2	MEIS1	MMP2	FXN	ATP5F1D
NONO	CALB1	MCCC2	RAN	EIF4A1	ANP32A	CYP2A6	ARL4A	SCYL1	AKT1	RPA1	WAPL
NAMPT	TXN	MCCC1	NOS2	MCM5	PROKR2	HCRTR1	NCOA7	TAC3	TET2	FDX1	ATP5MC3
RORB	PLAT	CAPZB	CRHR1	C1QBP	PSMA4	MTHFR	NXPH1	SELE	UCP1	RBP1	CIAO2B

CREM	MEF2C	ERO1A	CCNA2	EIF4A2	G0S2	CREB3	RASSF8	PLK4	CREB3L1	TBXT	S100G
NMU	ADCY10	RPE65	TFEB	MCM7	NFKB1	P2RX7	SPCS3	RRBP1	CYP8B1	ABCC1	MSH6
ADCYAP1	HERC2	NGF	KRAS	XRCC6	SDC3	PARP1	TANC2	RND1	RPS6KA5	HMOX2	BACE1
MTNR1B	CHEK1	PIK3R1	FBXO5	KRT10	RPS6KB1	HSD11B2	LCORL	NOTCH1	GCKR	RNF2	SMN2
AHR	SUMO1	THBD	CYP21A2	NXN	EIF4EBP1	STAG2	POF1B	DHFR	CYP1A2	SETD7	S100A1
PRL	EDN1	FAM50B	CAD	PPP2R2A	SCT	PEPC	TXNDC2	HIF1AN	SULT1A3	SREBF2	PTMA
CUL1	EIF2AK3	CDKN3	COPS8	RPN1	PTEN	INSL6	VTA1	MT3	RBL1	RPL4	SPARC
NCOR1	CAMK2G	AGT	DYNC1H1	SLC25A5	SMAD3	SOX14	BRMS1L	USP46	TIMP1	SLC27A1	UBE2N
METTL3	RPSA	TKT	TRPV1	TCP1	SNAI1	FBXL12	KCTD8	HCFC1	PTGS2	CXXC1	ASCL1
MAPK8	EEF2	NOS3	HDAC2	XRCC5	CD63	FBXL6	RHBDD1	RING1	PVALB	PSMD8	RAD18
SFPQ	CPNE8	HSPD1	SLC25A10	XRN2	GPD1	FBXL8	PELI2	SEC13	CDH1	COX11	TOPBP1
KAT2B	FLNA	HNRNPL	TOP2A	DDX20	TXNIP	FBXL22	ZNF662	DMAP1	CCKBR	H2AC6	RAD9A
MAGEL2	MCM3	GRIN2B	FBXO9	EFTUD2	KCNH4	ENSG00000265690	FERD3L	PSMD13	CYP11A1	CASP8	INTS7
SPSB4	CCT2	RPS3	FBXO22	KHDRBS1	BACH1	HSD11B1	MAP9	COX7B	HSPG2	EEA1	RAD1
ID3	CCT8	SOD1	ELANE	KRT2	LY96	ELF5	OR10R2	NUP88	ME1	NFE2L1	NABP2
MTA1	HNRNPA3	UBA1	FGF8	PABPC1	CLEC1B	IL1RN	HDX	RPS4X	RPL38	SLC22A1	ACKR1
PRKCG	HNRNPH2	CAND1	PHGDH	PCBP1	HK2	ADRA2A	CTXN3	MED30	PTS	GDF15	PTPRD
IMPDH2	CKAP4	GABBR1	ABCC2	PRDX4	TPT1	ALPP	CDC14C	MED10	IL1A	ATF7	CDC34
UBE3A	IGFBP1	PTPA	SDHA	PTBP1	PLIN1	TGFB1	ZBTB20	CCND1	EIF4G1	BATF3	CLDN1
CDK5	CSNK1G2	SERPINA6	ACLY	USP11	GRIN2C	TAT	BRF1	PRKACB	EIF3B	COP1	CSF1
HIF1A	UBA52	GNAS	MTHFD1	AKAP8	ACHE	AVPR1B	SCP2	PRKACG	TCF7L2	ANGPTL8	NEUROG1
PHLPP1	HNRNPA1	KDM2B	NDUFS3	CCT4	TSPO	CP	TSHB	PRKAR1B	BMP4	DLK1	HDAC9
NR0B2	G6PD	GAPDH	GMPS	GPRASP1	PDPK1	HNRNPDL	TCN1	PRODH	BRCA2	CALR	PLP1
ID1	PPP2R1A	CIRBP	EEF1A1	KPNA1	PHB2	ROCK1	TCAP	PRKAR2B	ABCC4	CSN2	PDE12
HCRT	CCK	ALAS1	ALDH9A1	MAP4	HFE	TWIST1	BSX	ADRB3	ALDH2	CDH13	GRIN2A
VIP	SENP1	TFRC	KHSRP	PABPC4	ETS1	ARHGDIA	JUN	DHCR7	LIPE	ADCY5	FN1
SKP1	PPARD	TIMP3	SMC4	CCT6A	RHO	NR1H4	GLUD1	SMC1A	GHSR	GLP1R	GRIN1
HNF4A	TRIM28	NOX4	ANAPC7	DDX17	CYP3A5	TLK2	TDO2	TAF1	DRD1	UBE2I	IRS1
INS	CTNNB1	COMT	HSPA1B	HNRNPF	RBCK1	KISS1	PSEN2	GNS	GNRHR	KMO	ADARB1
LEP	PIWIL1	BVES	BAG2	HNRNPM	ADH4	HSP90AB1	RBX1	NFKBIA	MRGPRX1	PNMT	MAP2K5
CA6	NPY5R	CSE1L	SMC2	ILF3	SF3A3	TPTEP2-CSNK1E	GSK3A	CAMK2D	SCN5A	BAG1	NEFH
DDB1	EGR3	CHEK2	SRM	DDX3Y	LDHA	ENHO	OPRL1	KMT2C	SLC6A2	G6PC2	AKAP13
KLF10	CACUL1	PPP2R5E	DNAJC7	DHX15	TUBB3	ACTL6A	PNOC	SMARCD1	DDB2	ACER2	RAD17
KAT5	FBXL15	ACTN1	ATP5F1A	SCYL2	GSTM3	CTCF	NR4A1	ACBD3	CSNK1G3	HEBP2	SIRPA
RBM4B	RPS6	KRT17	MARS1	GARS1	LDHB	ZNF704	ID4	RFX4	POU1F1	SLC48A1	CD47
USP9X	CD36	GFPT2	DARS1	GEMIN5	IDI1	IL2RA	CYP2C19	PPP4C	TRIM24	RHNO1	CUL4B
PIWIL2	PSMD2	DDX3X	DCTPP1	PABPC3	LDHC	TNRC6B	YY1	SETD2	FOSL2	BOK	GNAZ
SIRT6	CPT1A	CCT5	EPRS1	SEC16A	UGP2	GCH1	KCNH7	HNMT	RBM3	RAB27A	VAMP7
KLF15	UBC	PKM	IARS1	PPP2R2D	VAPA	TYMS	FKBP5	MFSD2A	USP21	PAX6	NES
IL6	NTS	GSN	RARS1	OBI1	AZIN1	TPH2	GFAP	IDH1	NQO2	MGMT	UBE2K
TNF	PSMC3	ACTN4	GCN1	CACNA1C	HLA-DMA	OXT	AHCY	ATF5	PAH	ACTB	AMBRA1
MAPK10	PSMC5	MYH14	CYP7A1	GC	HERPUD1	ATF6	HCN2	IL2	MAT1A	ECE1	DCAF8
MAPK9	PSMD3	DDOST	ERC2	PTGDS	PPP1R3C	DIO2	BLZF1	APOA4	MAT2A	STXBP1	WDR61
SIK1	PSMD11	FLNC	DEFB1	SYVN1	IFNG	PDC	SLC24A3	RARG	QDPR	CPS1	CEBPZ
VIPR2	PSMC6	EZR	CEBPB	RRH	DUSP1	VWF	PLAU	THRA	MECP2	CYP2C8	DCAF7
HUWE1	PSMD1	KRT6A	CYP2D6	SCN8A	OXTR	ADCYAP1R1	MEN1	MYOD1	ST3GAL3	GALNS	INTS3
ASS1	PSEN1	PLEC	CHKA	LMAN1	TRRAP	PFAS	DROSHA	G6PC1	RAPGEF3	MAP2K3	NOL7
DDA1	DCAF11	DET1	PTPDC1	H2BC12L	MIR6883	BTBD9	CARTPT	CDK4	CHRM1	CHRNB2	CLDN4
CRX	DDX5	DHX9	DYRK1A	FAS	GHRH	HNF1B	HOMER1	KCND2	KCNMA1	LGR4	MIR1281
MIR3064	MIR4751	MIR5047	MIR5572	MTTP	NLGN1	NPS	NTRK1	NTRK3	OPN3	PAX4	PRF1
PRKAA1	PRKG2	PROK1	ROCK2	SETX	SFTPC	SIX3	UTS2	UTS2R	ZFHX3	ELOVL3	DELEC1
CSF2	GHRHR	FBXL21P	TIMELESS	HNRNPD	MYBBP1A	KCNA2					
